# Immunophenotyping and Efficacy of Low Dose ATG in Non-Sensitized Kidney Recipients Undergoing Early Steroid Withdrawal: A Randomized Pilot Study

**DOI:** 10.1371/journal.pone.0104408

**Published:** 2014-08-11

**Authors:** Monica Grafals, Brian Smith, Naoka Murakami, Agnes Trabucco, Katherine Hamill, Erick Marangos, Hannah Gilligan, Elizabeth A. Pomfret, James J. Pomposelli, Mary A. Simpson, Jamil Azzi, Nader Najafian, Leonardo V. Riella

**Affiliations:** 1 Department of Transplant Surgery, Lahey Clinic Medical Center, Burlington, Massachusetts, United States of America; 2 Department of Medicine, Georgetown University, Washington, D.C., United States of America; 3 Transplantation Research Center, Renal Division, Brigham & Women's Hospital, Harvard Medical School, Boston, Massachusetts, United States of America; 4 Department of Medicine, Beth Israel Medical Center, New York, New York, United States of America; 5 Department of Nephrology, Cleveland Clinic Florida, Weston, Florida, United States of America; University of California Los Angeles, United States of America

## Abstract

Rabbit antithymocyte globulin (ATG) is commonly used as an induction therapy in renal transplant recipients, but the ideal dosage in tacrolimus-based early steroid withdrawal protocols has not been established. The purpose of this pilot study was to determine the immunophenotyping and efficacy of lower dose ATG in low immunological-risk kidney transplant recipients. In this prospective study, 45 patients were randomized (1∶1) to our standard dose ATG (total dose 3.75 mg/kg)(sATG) vs. lower dose 2.25 mg/kg (lowATG). All patients underwent early steroid withdrawal within 7 days. The primary end point was biopsy-proven acute rejection at 12 months. Prospective immunophenotyping of freshly isolated PBMCs was performed at baseline, 3, 6, 12 months post-transplant. The rate of acute rejection was 17% and 10% in the sATG and lowATG, respectively. Effector memory T cells, Tregs and recent thymic emigrants T cells had similar kinetics post-transplant in both groups. No statistically significant differences were found in graft survival, patient survival or infections between the two groups, though there was a non-significant increase in leukopenia (43%v s. 30%), CMV (8% vs. 0) and BK (4% vs. 0) infections in sATG group vs. lowATG. In sum, in low immunological risk kidney recipients undergoing steroid withdrawal, low dose ATG seems to be efficacious in preventing acute rejection and depleting T cells with potentially lower infectious complications. A larger study is warranted to confirm these findings.

**Trial Registration:**

ClinicalTrials.gov NCT00548405

## Introduction

Most kidney transplant centers in the United States utilize induction agents as part of their immunosupression protocols as it is believed that these agents prevent early acute rejection episodes and possibly improve graft survival [1]–[3]. Rabbit antithymocyte globulin (ATG or Thymoglobulin) is the most common induction agent used in renal transplantation and is used in more than 55% of kidney transplants in the USA, despite not being FDA-approved for this use [2]. ATG causes T-cell depletion by inducing complement-dependent cell lysis. It also modulates cell surface and adhesion molecules that regulate T-cell function and leukocyte endothelial interactions, respectively [1], [4], [5]. Induction therapy with ATG results in a lower acute rejection rate, but is also associated with a higher risk of infections and malignancies [6]–[8]. An ATG dosage of 6–10 mg/kg has traditionally been the standard of care in most transplant centers in the USA [5], [9]–[12]. ATG has been widely studied in the cyclosporine and low-dose steroid maintenance era, however the optimal dosage of ATG in the setting of tacrolimus use and steroid withdrawal has not been determined.

Our transplant center utilizes 3.75 mg/kg of ATG as induction in both deceased and living donor renal transplantation. Because of drug acquisition costs, risks of malignancies and infections, it is imperative to find the minimum effective dose of ATG that still allows for minimization of maintenance immunosupression. Moreover, the expansion of steroid withdrawal regimens urges a reassessment of optimal ATG dosage. Our hypothesis is that lower ATG dose is effective and safe in non-sensitized renal transplant recipients undergoing steroid withdrawal. Herein, we report an open-label, single-center prospective randomized pilot study comparing two lower dosage ATG regimens (3.75 mg/kg vs. 2.25 mg/kg) in low immunological risk recipients who underwent steroid withdrawal and maintenance immunosuppression with tacrolimus and mycophenolate mofetil.

## Methods

### Study design and patient selection

This is a prospective, open-label, single center randomized pilot study in adult renal transplant recipients at Lahey Clinic Medical Center, Burlington, MA. Patients above 18 years of age who were scheduled for a living or deceased donor renal transplant were eligible for enrollment. Exclusion criteria included a multi-organ transplant; a current or historic panel reactive antibody >20%; presence of donor specific anti-HLA antibodies; and contraindication to ATG use. The recruitment time was pre-established for 2 years with one additional year of follow-up. The protocol for this trial and supporting CONSORT checklist are available as supporting information (see Checklist S1 and Protocol S1).

### Ethics statement

The study protocol was reviewed and approved by an Institutional Review Board at Lahey Clinic Medical Center and was conducted in full conformance with the principles of the Declaration of Helsinki. All study participants provided signed informed consent previous to randomization. The study is listed on http://clinicaltrials.gov (NCT01280617).

### Randomization and treatment

**Figure 1:**
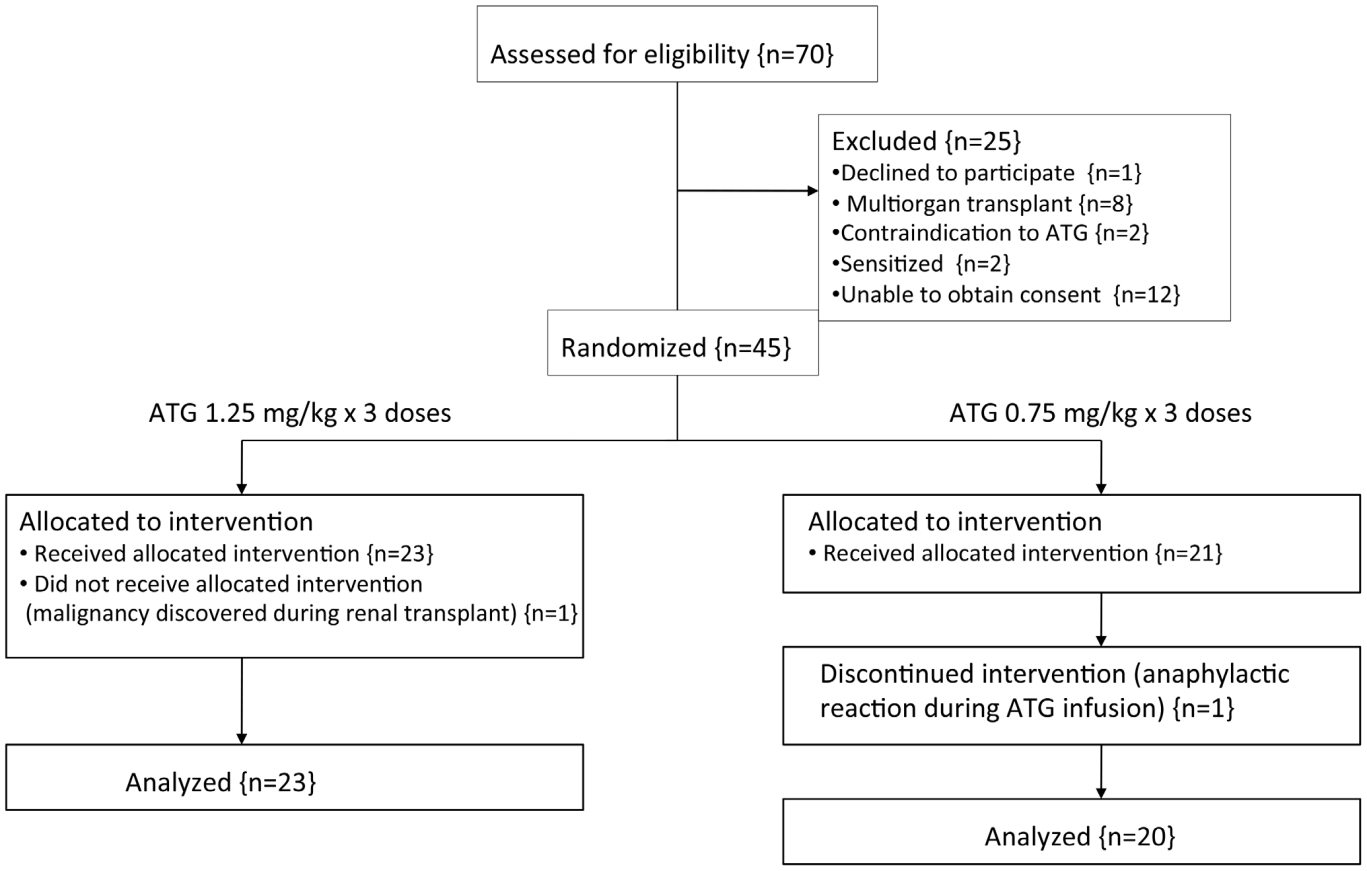
Flow diagram of patients screened and enrolled in the study.

After the screening process, eligible patients were randomized in a 1∶1 ratio either to receive standard dose ATG of 1.25 mg/kg×3 doses (3.75 mg/kg total dose) [sATG] or low dose ATG of 0.75 mg/kg×3 doses (2.25 mg/kg total dose) [lowATG] (Figure 1). For obese patients, the maximum weight used in the dose calculation was 100 kg. After patient enrollment by a transplant nephrologist, computer-generated protocols were utilized for randomization (performed by research coordinator and implemented by transplant nephrologist).

Patients on both arm received ATG intraoperatively on day 1, followed by daily administration of ATG on days 2 and 3, at the dosage according to the assignment.

All patients received maintenance immunosuppression with tacrolimus, mycophenolate mofetil (MMF) and a 7-day corticosteroid taper. Tacrolimus was initiated on day 1 at 2 mg twice a day and target blood concentrations were 8–10 ng/ml for the first 6 months post-transplant. MMF was dosed as 1000 mg twice a day in both groups. Methylprednisolone was given intravenously at 500 mg on day 0, 200 mg on day 1 and 100 mg on day 2. Prednisone 20 mg was given by mouth on day 3 and 10 mg was given on day 4, followed by fast taper. No further corticosteroid use was planned after the first post-operative week unless patients were on long-term prednisone prior to transplantation for other reasons.

All patients received trimethoprim 80 mg-sulfamethoxazole 400 mg administered orally daily for six months for bacterial and *Pneumocystis jiroveci* prophylaxis. Inhaled pentamidine monthly for 6 months was given in patients allergic to sulfa products. Valganciclovir 450 mg daily or adjusted dose for renal function was given for cytomegalovirus (CMV) prophylaxis for three months in low-to-intermediate risk patients (seropositive donor and recipient [D+/R+], seronegative donor and recipient [D−/R−] and seronegative donor and seropositive recipient [D−/R+] and for 6 months in high-risk seropositive donor and seronegative recipient [D+/R−] patients.

### Endpoints

The primary endpoint was the incidence of biopsy-proven acute rejection (BPAR) at 12 months post-transplant. This was determined by pathological evidence of rejection defined by the Banff ‘97 criteria. Delayed graft function was defined as the need for dialysis within seven days after transplantation. Leukopenia was defined as a WBC count of 3,500 cells/mm^3^ or less. Secondary endpoints included: patient survival, graft survival and function (serum creatinine at 3, 6, 12 month), incidence of infection, leukopenia (defined as WBC<3,500) and cancers.

### Peripheral blood mononuclear cells (PBMC) isolation

Peripheral blood samples were obtained from patients pre-transplantation, at 3, 6 and 12 months post-transplant. Samples were all collected between 7–9am and were processed within 4 hours at the Immunological Core Facility at the Transplant Research Center, Brigham and Women's Hospital. PBMCs were isolated by density gradient centrifugation using Ficoll-Paque solution (GE Healthcare Biosciences) spun at 800 *g* for 30 minutes at 20°C.

### Flow cytometric analysis

Freshly prepared PBMCs were used for flow cytometric analysis using four-color flow cytometry on a FACS Calibur analyzer (Becton Dickinson, NJ) with cell surface staining using anti-CD45RO-FITC, anti-CD31-FITC, anti-CD25-PE, anti CD4-PerCP, anti-CD4-APC, anti-CD8-PerCP, anti-CD62L-APC, anti-CD45RA-APC (BD Biosciences, San Jose, CA, USA). For intracellular staining of FoxP3, permeabilization solution (eBioscience, San Diego, CA, USA) and anti-Foxp3-FITC (eBioscience, San Diego, CA, USA) were used based on manufacturer's protocol.

### Statistical analysis

For this pilot study, we estimated that 40 participants in total were needed [13]. Statistical analysis was performed in GraphPad Prism version 5.0d and Microsoft Excel 2011. Continuous variables were analyzed using two-tailed *t* tests if normally distributed, while nonparametric Mann-Whitney test was used if variable was not normally distributed. Categorical variables were analyzed using chi-square testing, unless expected frequencies were less than five in any group, in which case Fisher's exact test was used. We report means ± SDs. P-values less than 0.05 were considered significant. With the exception of patient survival, all clinical outcomes were censored (patients not followed) beyond the date of graft failure. Thus, analysis of renal function (serum creatinine and eGFR) at various times post-transplant were based on comparing patients who were still alive with functioning grafts at those times.

## Results

### Study Patients

From the initiation of trial recruitment in November 2010 until the completion of recruitment in April 2013, seventy patients were assessed for eligibility for the study and forty-five patients underwent randomization (Figure 1). Data collection continued until August 2013 with a mean follow-up of 15 months. The two arms were well balanced with no major differences in terms of living versus deceased donor transplant, recipient's gender, age, ethnicity, re-transplant, diabetes, CKD etiology, dialysis time prior to transplant, panel reactive antibody (PRA), donor age and kidney weight (Table 1), with the exception of a slightly higher prevalence of coronary artery disease in the lowATG group compared with sATG (n = 9 vs 4, p = 0.06).

**Table 1:** Baseline characteristics of renal transplant recipients and donors.

	sATG 3.75 mg/kg (n = 23)	lowATG 2.25 mg/kg (n = 20)	P Value
**Recipients' characteristics**			
Age at transplant (yr)	52.9±12.1	56.6±11.6	0.311
Body weight (kg)	78.5±22.3	88.5 ±21.8	0.148
Body mass index (kg/m^2^)	27.6±4.8	29.1±5.9	0.364
PRA (%)	0	0	
Pre-transplant diabetes (%)	11 (47.8)	9 (45)	0.874
Re-transplant (%)	2 (8.6)	1 (5.0)	0.499
Race/ethnicity (%)			0.246
White	13 (56.5)	16 (80)	
Hispanic	6 (26.0)	2 (10)	
African American	2 (8.7)	2 (10)	
Other	2 (8.7)	0 (0)	
Gender (%)			0.456
Males	16 (69.5)	16 (80)	
Females	7 (30.4)	4 (20)	
CKD etiology (%)			0.859
DM	11 (47.8)	9 (45)	
HTN	1 (4.3)	3 (15)	
GN	5 (21.7)	6 (30)	
PKD	4 (17.3)	1 (5)	
Other	2 (8.7)	1 (5)	
Length of dialysis prior to transplant (months)	24.7±23.9	31.2±24.7	0.389
History of CAD	4 (17.4)	9 (45)	0.0591
Average follow up (months)	15.9±6.5	17.8±5.15	0.796
			
**Donors' characteristics (%)**			0.392
*Females*	10 (43.5)	6 (30)	
*Males*	13 (56.5)	14 (70)	
Type of donors (%)			0.849
*Deceased donor*	14 (60.8)	12 (60)	
*Living donor*	9 (39.2)	8 (40)	
Extended-criteria donor	4 (17.4)	6 (30)	0.349
Donor age (yr)	44.4±13.4	47.5±13.5	0.468
CIT (min)	378±332	433±421	0.688
Kidney weight (grams)	213.4±53.5	209.1±51.0	0.809

CKD, chronic kidney disease; CAD, coronary artery disease; CIT, cold ischemia time; GN, glomerular nephritis, ECD, extended-criteria donor; PKD, polycystic kidney disease; PRA, panel reactive antibody.

### Outcomes

**Figure 2:**
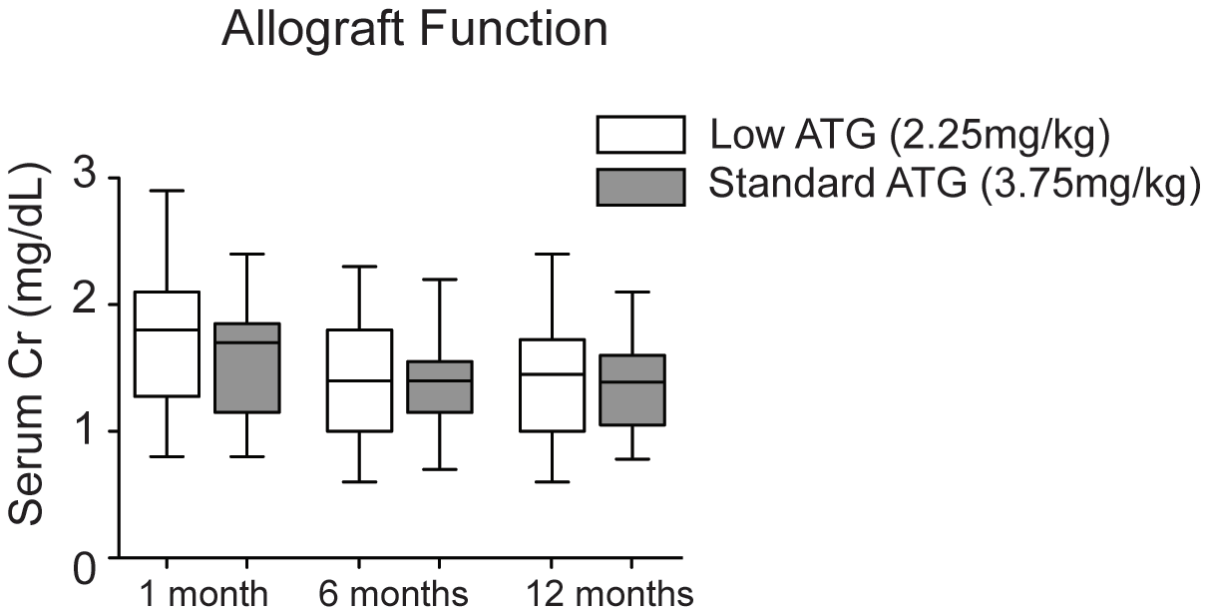
Graft function after transplantation. Serum creatinine was measured at 1, 6, 12 months post-transplantation, as a surrogate of graft function. Means±SD. At each time point, there was no statistically significant difference in serum creatinine between low-dose and high-dose arms by unpaired *t*-test analysis.

The two arms showed no significant difference at the primary endpoint of biopsy-proven acute rejection (BPAR). The rate of acute rejection in the lowATG group was 10% compared to 17% in the sATG group (p = 0.66) (Table 2). Patient survival at 1 year in the lowATG group was 85% compared with 100% in the sATG (p = 0.11) (Table 2). There were two patient deaths in the lowATG group related to cardiovascular events with a functioning graft. Both patients had been on hemodialysis for more than five years prior to transplantation, had history of coronary artery disease and diabetes, and were long-term smokers. The etiology of the third death was undetermined. Delayed graft function occurred in 40% of patients in the lowATG group compared with 14.3% in the sATG group (p = 0.05). Serum creatinine (sCr) and eGFR were similar between both groups at 1, 6 and 12 months post-transplant (Figure 2). At 12 months post-transplant, the serum creatinine in the lowATG was 1.4±0.44 mg/dl compared with 1.33±0.0.36 in the sATG group (p = 0.56). There were no primary graft failures on either group. There was no significant difference in the tacrolimus trough levels and mycophenolate dosing between the two groups (Table 2).

**Table 2:** Outcomes and complications after kidney transplantation.

	sATG 3.75 mg/kg (n = 23)	lowATG 2.25 mg/kg(n = 20)	P Value
**Rejection**	4 (17%)	2 (10%)	0.662
**Leukopenia**	10 (43%)	6 (30%)	0.342
**Severe infection**	3 (13%)	2 (10%)	0.556
**BK nephropathy**	1 (4%)	0 (0)	0.331
**CMV infection**	2 (8%)	0 (0)	0.487
**Patient survival**	23 (100%)	17 (85%)	0.106
**Delayed graft function (DGF)**	3 (14.3%)	8 (40%)	0.0508
**sCre post-transplant (mg/dL)**			
*1 month*	1.58±0.10	1.77±0.13	0.267
*6 months*	1.38±0.08	1.41±0.10	0.853
*12 months*	1.33±0.07	1.40±0.10	0.563
**Trough tacrolimus level** (mean±SD; ng/mL)			
*3 months*	7.24±1.50	7.78±2.56	0.421
*6 months*	7.60±1.80	6.81±1.99	0.237
*12 months*	7.20±2.62	5.67±4.05	0.204
**Mycophenolate Mofetil dose** (mean±SD; mg/day)			
*3 months*	1730±438	1433±457	0.092
*6 months*	1535±458	1384±463	0.402
*12 months*	1354±482	1181±462	0.392

CMV, cytomegalovirus; sCre, serum creatinine.

### Adverse events

We observed more leukopenia in the sATG group compared with the lowATG group (43% vs. 30%, p = 0.34) (Table 2). There were two cases of CMV infection in the sATG group and one of these patients also developed BK infection. There were no cases of either BK or CMV in the lowATG group. The incidence of severe infection requiring hospital admission was 13% in the sATG group compared to 10% in the lowATG group (p = 0.55). There was one case of squamous cell cancer on each group and one of prostate cancer in the lowATG group. There was no case of post-transplant lymphoproliferative disorder, Kaposi sarcoma or renal cell carcinoma.

### Effects of ATG on peripheral T cells

**Figure 3:**
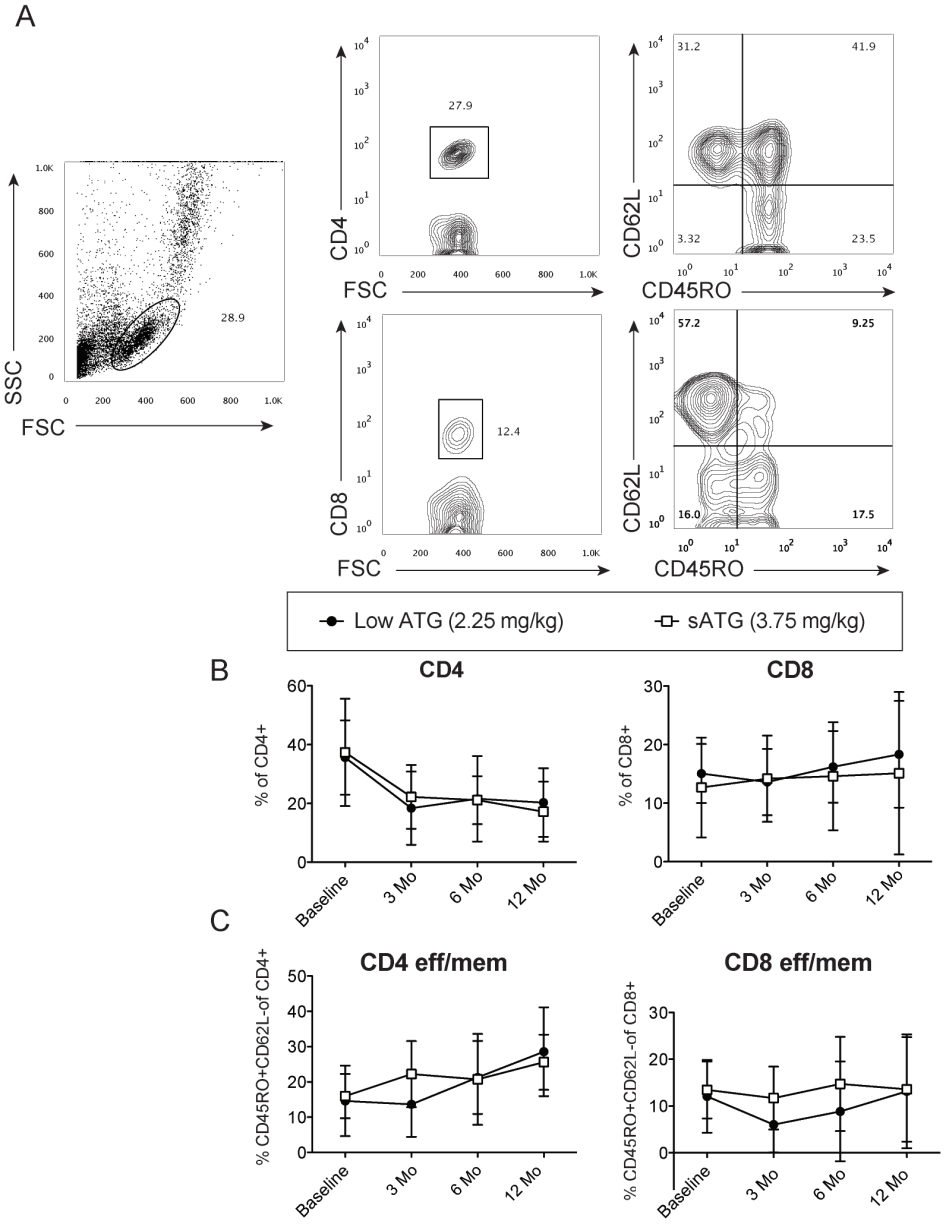
Flow cytometric analyses of peripheral T cells at different time points after transplantation. A, Representative gating strategy of peripheral blood mononuclear cells with live gate, CD4 and CD8 subsets and effector memory T cells (CD45RO^+^CD62Llow). B, Percentage of total CD4 cells and total CD8 cells at 0, 3, 6 and 12 months after transplantation. C, Percentage of CD4^+^ and CD8^+^ effector memory cells after transplantation. Data are expressed as mean and standard deviation (n = 18-20 per group).

**Figure 4:**
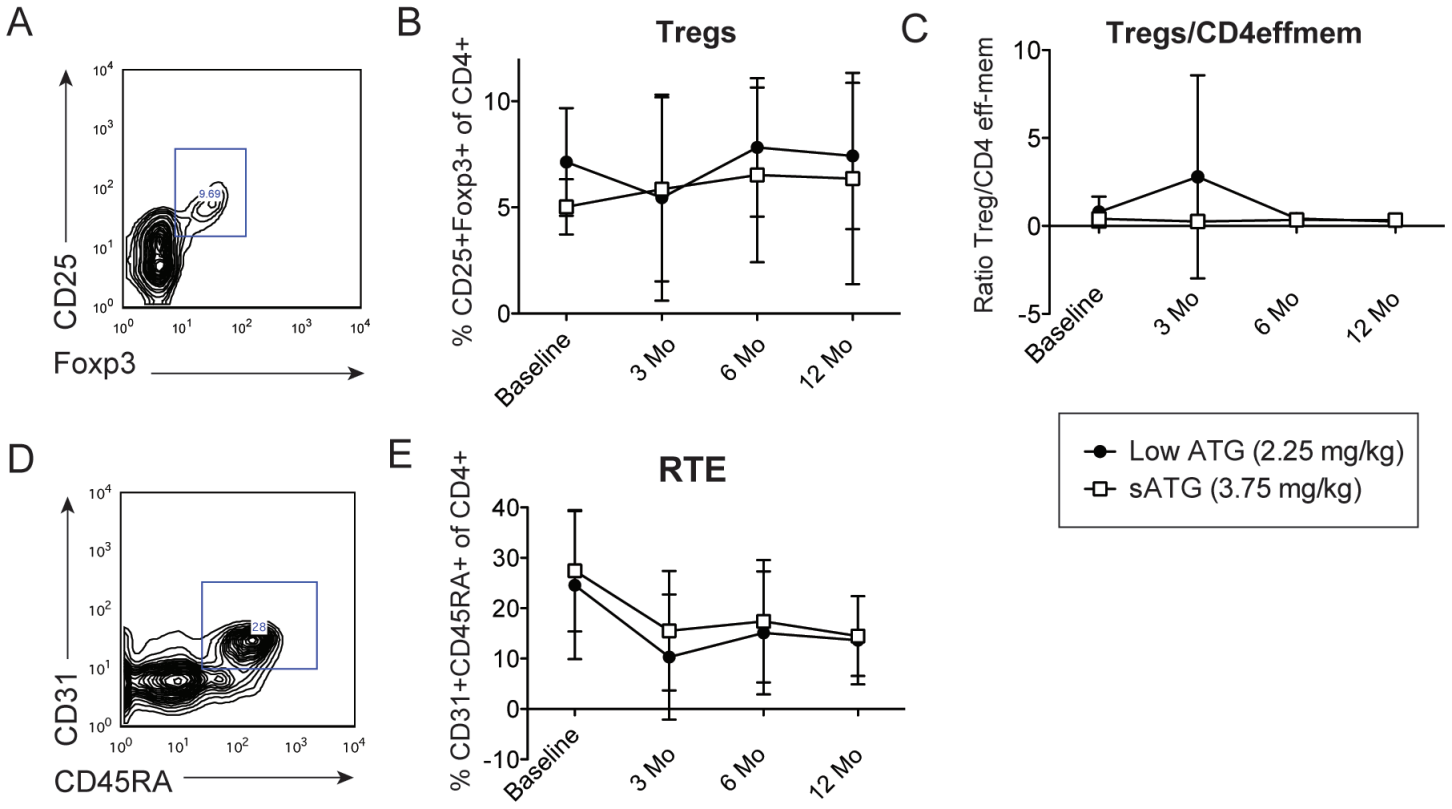
Flow cytometric analyses of Tregs and recent thymic emigrants (RTE) CD4 cells at different time points after transplantation. Representative contour plots of Tregs (A) (CD25^+^Foxp3^+^ of CD4^+^ cells) and RTEs (D) (CD45RA^+^CD31^+^ of CD4^+^ cells) cells after gating on CD4^+^ T cells at different time points after transplantation. Percentage of Tregs (B) and ratio of Tregs related to baseline (C) at different points after transplantation. E, Percentage of RTEs at different points after transplantation. Data are expressed as mean and standard deviation (n = 18-20 per group).

We compared the kinetics of peripheral blood T cell subsets at baseline and at 3, 6 and 12 months post-transplant between groups (Figure 3, 4). While CD8 T cells were recovered close to baseline after 3 months on both groups, CD4 T cells were persistently lower in both groups up to 12 months post-transplant (20±7.1% lowATG vs. 17±3.6% high ATG compared to baseline 35±3.6% and 37±5.5%, respectively) (Figure 3B). Overall, the percentage of effector memory T cells (CD45RO+CD62Llow) was reduced at 3 months after transplantation but increased after that, reaching its highest percentage at 12 months (Figure 3C). Tregs' percentage slightly increased post-transplant in both groups, though the standard deviations were wide and differences non-significant (Figure 4B).

The ratio of Treg/CD4effmem percentage has been suggested to indicate immune regulation and potentially less risk of rejection [14]–[16]. We observed a trend towards higher Treg/CD4effmem at 3 months after transplant in the lowATG group, however ratio was similar to baseline at subsequent time points (Figure 4C). We could not identify an association between lower ratio of Treg/CD4effmem and later rejection (data not shown). Recent thymic emigrants (RTE) are a subset of T cells that proliferate in response to IL-7 and in the absence of other costimulation, a property that may help in the reconstitution of T cells after T-cell depletion with ATG [17]. These cells are characterized by the expression of CD4, CD31 and CD45RA. We assessed the kinetics of RTEs after T cell depletion; the percentage of RTEs significantly decreased after ATG on both groups and did not recover back to baseline after that (Figure 4D, E). We did not observe any correlation between the percentage of RTE and the development of leukopenia after ATG.

### Cost minimization

Though beyond the scope of this paper, the cost-effectiveness of the different strategies was assessed. ATG costs $600 per 25 mg vial. On an average 80 kg representative patient, the standard dose ATG recipient (3.75 mg/kg total) received 100 mg×3 doses (12 vials), which equals $7,200, while the lowATG recipient (2.25 mg/kg total) received 50 mg×3 doses (6 vials) with a total cost of $3,600. Taking into account the individual weight of patients on this study, the average cost saving was approximately U$2,307 dollars per patient in the lowATG arm compared to sATG.

## Discussion

ATG is a potent immune-depleting induction therapy that has significantly reduced the rate of acute rejection in solid organ transplantation. At the same time, however, high dose ATG is associated with increased incidence of leukopenia, malignancy and infectious complications [6]–[8]. Despite more than 20 years of clinical use, its optimal dose is still controversial, in particular in the setting of steroid withdrawal.

For the evaluation of efficacy and safety, multiple trials have been conducted to optimize ATG dosage [18]. The initial trials used relatively high cumulative doses of ATG of 10.5 mg/kg with cyclosporine and low-dose steroids maintenance regimen [19], [20], followed by efforts to reduce the dosage of ATG. Recently published observational study using ATG induction registry of 2,322 living donor kidney transplants from 49 different centers reported that the mean cumulative dose was 5.29 mg/kg, with 93.6% rejection free survival at 5 years. The registry consisted of a heterogeneous group of patients and the majority of them were receiving low dose steroids long-term [21], leading to difficulties in extrapolating the results to this specific subgroup of patients. Another recent 10-year follow-up study using an early steroid withdrawal reported the use of a total dose 6.25–7.5 mg/kg of thymoglobulin [22]. Wong *et al*. compared different cumulative dosages of thymoglobulin (3.0 mg/kg vs 4.5 mg/kg), and showed no statistically significant difference in acute rejection rate and graft function up to 24 months post transplantation [23]. Though it was relatively small study (7 vs. 9 patients in each arm) and all patients were continued on steroids, this encouraged us to further reduce thymoglobulin dosage in our transplant program.

Our study showed that a lower dose of 2.25 mg/kg of ATG was safe and effective in preventing acute rejection in combination with early steroid withdrawal in our non-sensitized kidney transplant cohort. This dose was about 1/3 of the commonly used dose of ATG in most centers. Two cardiovascular deaths with functioning grafts in the lowATG group were likely to have occurred by chance despite randomization, and were unlikely related to difference in ATG dose given. The barely significant increase in CAD history in the low ATG group also supports this hypothesis. In addition, we observed a non-significant reduction in the incidence of leukopenia, CMV and BK infections in lowATG arm, suggesting possible benefit from lower dose ATG. In contrary, DGF was more common in the lowATG group (p = 0.05). Reviewing Table 1, we observe a non-significant trend towards higher proportion of ECD kidneys and greater mean cold ischemia time in the lowATG group (30% vs 17% and 433 vs 378 min, p>0.05). Since prior data from larger randomized trials did not show lower DGF in ATG compared to other induction therapies (18), it is hard to conclude the significance of these findings in this smaller cohort. Despite that, the absence of primary graft losses on both groups and the good renal function at 12 months are reassuring. Although this report is an exploratory data with small number of cases, these are encouraging results that must be further validated in a larger trial. Since study conclusion, Lahey Clinic has now adopted the lower dose ATG as standard of care in non-sensitized transplant recipients.

Aside from ATG, other induction agents such as basiliximab (anti-IL2 receptor mAb, Novartis) or alemtuzumab (anti-CD52 mAb, Berlex Laboratories) have been used in combination with tacrolimus maintenance and steroid withdrawal. One of the largest prospective trials on steroid withdrawal reported that the rejection rate was higher with basiliximab compared to ATG (24.2% vs 14.4%), though long-term graft outcomes were similar [24]. Alemtuzumab has been tested on a recent randomized controlled trial in patients undergoing steroid withdrawal and was found to be associated with lower rates of acute rejection when compared to basiliximab in low-risk recipients, though late rejections were more common in the alemtuzumab group (1–3 years after transplant) [25]. This is a concern since the follow-up of transplant recipients tends to be less frequent after the first year of transplantation and a rejection episode may go unnoticed for few months.

In face of the increased budget constraints that transplant centers and hospitals are undergoing and the huge expense that ATG accounts for, reduction of ATG dose could have an important financial benefit and a possible cost-reduction of ∼U$3,000/patient could be obtained by reducing the ATG dose from our standard protocol (3.75 mg/kg total) to the study dose of 2.25 mg/kg total. Although basiliximab is less expensive (∼$4,000 per patient) than ATG, it is a relatively less potent immunosuppressant and is associated with a higher rate of rejection in patients that undergo steroid withdrawal [24]. Alemtuzumab costs less as well ($1,689 for the recommended single dose of 30 mg) and requires shorter hospital stay, given its use as a one-time dose at the time of transplant. However, alemtuzumab is associated with significantly more prolonged T cell depletion, which may require years to recover [18], [26]. In addition, Alemtuzumab may promote the development of donor-specific antibodies through its significant effect on B cells subsets [27]. Nevertheless, thymoglobulin and alemtuzumab had similar patient and graft outcomes in recent randomized trials [28], [29]. In sum, ATG seems to be the preferred induction agent for patients undergoing steroid withdrawal.

Immune reconstitution after administration of T-cell depletion by thymoglobulin is also of great interest, in particular with experimental evidence suggesting that it may promote tolerance through the induction and expansion of Tregs [14], [30]. In children, thymopoiesis plays an important role in the reconstitution of T cells after ATG depletion, while in adults, homeostatic proliferation is dominant due to the progressive atrophy of thymus with age [30], [31]. This homeostatic proliferation is predominantly an IL-7 mediated process, enabling T-cell clone which survived depletion to expand. Recent thymic emigrants (RTE) are a subset of T cells that proliferates in response to IL-7 in the apparent absence of other stimulation and may play an important role in the lymphocyte reconstitution after T-cell depletion [17]. Our findings suggest that both higher and lower dose ATG significantly reduce the RTE subset and this subset remains lower than at baseline even 12 months following transplantation, though there was a trend towards greater reduction in the sATG group. This may allude to some long-term consequences of ATG in the homeostasis of T cell subsets and using a lower dose may have potential to limit that. It is clear that immunosuppression has this cumulative effect and once given, it is not possible to take it back and long-term consequences in the immune system are seeing decades to come. In face of our overall prolongation of survival, it is critical to minimize the life-immunosuppression dose.

The limitations of our study include the small number of patients, the predominance of Caucasians, the lack of blinding, the absence of protocol biopsies and the exclusion of sensitized recipients. A longer follow up would also be necessary to fully elucidate the impact of ATG dose on the malignancy rate.

In conclusion, low-dose ATG (2.25 mg/kg) is a promising option as an induction therapy in low-risk patients with no significant differences in acute rejection or graft function when compared to sATG (3.75 mg/kg), and with possibly less leukopenia and infectious complications. In addition, our peripheral blood cell analyses showed similar T cell depletion efficiency with the lower dose ATG. Larger studies are warranted to confirm these findings.

## Supporting Information

Checklist S1 **CONSORT checklist.**
(DOC)

Protocol S1 **Trial protocol.**
(DOC)
