# Supplementary material for: Immunophenotyping and Efficacy of Low Dose ATG in Non-Sensitized Kidney Recipients Undergoing Early Steroid Withdrawal: A Randomized Pilot Study
Source: PLoS One. 2014 Aug 11;9(8):e104408. doi: 10.1371/journal.pone.0104408 (PMC4128673; doi:10.1371/journal.pone.0104408)
Supplement: Protocol S1 — Trial protocol. (DOC) [file pone.0104408.s002.doc]

**
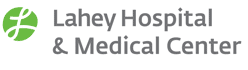
**

**LAHEY CLINIC, INC.**

**DIVISION OF RESEARCH**

**Research Consent Form (DR-2)**

**Principal Investigator: Hannah Gilligan, MD LCID: 2010-065**

**Protocol Title: LOW DOSE THYMOGLOBULIN AS INDUCTION AGENT IN PREDNISONE-FREE REGIMENS OF RENAL TRANSPLANT RECIPIENTS**

**Tel: (781) 744- 2500**

**INTRODUCTION**

You are being asked to participate in this research study because you will have a renal transplant. You may discuss this treatment with your health care team. Your doctor will answer any questions you may have.

Your participation in this clinical trial is voluntary and your refusal to participate will not affect any medical care or benefit to which you would otherwise be entitled. If you decide to participate, you will be provided with a copy of this signed form. You will be told of any significant new findings during the course of this study and you are free to withdraw your consent and discontinue participation at any time. The Robert Wise Grant is providing funding for this study.

A description of this clinical trial will be available on [http://www.ClinicalTrials.gov](http://www.ClinicalTrials.gov/), as required by U.S. Law. This Web site will not include information that can identify you. At most, the Web site will include a summary of the results. You can search this Web site at any time.

**WHY IS THIS STUDY BEING DONE?**

The purpose of this study is to test the effectiveness, safety and side effects of low dose thymoglobulin as an induction agent (this is a medication that is given to you at the time of the renal transplant to decrease your immune response against the new renal transplant that you will have). Renal transplants last the same amount of time as 20 years ago even though new medications have been developed. One of the main culprits of the decreased life of renal transplants is infection, in particular BK nephropathy which is a virus that takes advantage of the immunosupressed renal transplanted recipient and attacks the transplanted kidney causing renal scaring and on many occasions renal failure.

The current standard of care includes giving thymoglobulin 1.0-1.5mg/kg of body weight for 3-7 doses to renal transplant recipients at the time of transplant. There have been some studies that have shown that it may be advantageous to try lower doses of thymoglobulin for renal transplant recipients.

## HOW LONG WILL I BE IN THE STUDY?

The total time for the treatment part of the study is approximately12 months. You will be followed indefinitely for your renal transplant.

**HOW MANY PEOPLE WILL TAKE PART IN THE STUDY?**

Approximately 100 patients will participate in this study at the Lahey Hospital & Medical Center and its affiliates as applicable.

**WHAT IS INVOLVED IN THE STUDY?**

You will be randomized to either our standard of care thymoglobulin dose (1.25mg/kg for 3 doses) or lower thymoglobulin (0.75mg/kg for 3 doses). There will be sealed envelopes to perform the randomization process. Half of the envelopes will say 1.25mg/kg dosing and the other half of the envelopes will say 0.75mg/kg dosing. We will sequentially choose the sealed envelopes at the time of the randomization process.

**BEFORE YOU BEGIN THE STUDY:**

**DURING THE STUDY:**

You will not need any extra clinic visits as part of this study.

**WILL EVERYONE RECEIVE THE SAME TREATMENT AND HOW WILL IT BE DETERMINED?**

Before starting treatment, you will be assigned to one of two treatment groups: either standard of care thymoglobulin vs low dose thymoglobulin (often referred to as a “treatment arm”). Randomized means that you are assigned to a treatment group (or arm) by chance. Neither you nor your doctor can choose the group you will be in.

**WHAT ARE THE FORESEEABLE RISKS AND SIDE EFFECTS TO THIS STUDY?**

We do not know if there will be a higher rate of rejection in the low dose thymoglobulin group, neither do we know if the standard dose thymoglobulin group will have a higher risk of infection.

**DISCLOSURE**

For your safety, you must disclose to the study doctor all of your past and present diseases and allergies of which you are currently aware. You must also share with the study doctor all prescription medications, over-the-counter medications (e.g., Tums, cough syrup, vitamins and minerals, etc.), herbal preparations (e.g., St. John's Wort, ginkgo biloba, ginseng, Melatonex, etc.), and nutritional supplements (e.g., Ensure, power bars, etc.) that you are currently taking, since it is not fully understood how any of these drugs, preparations, and supplements interact with one another in every case.

**ARE THERE BENEFITS TO TAKING PART IN THIS STUDY?**

Taking part in this study may or may not make your health better. While your doctor hopes that this study regimen will be useful in improving long term survival of renal transplants there is no proof of this yet. Information learned about the treatment(s) used in this study may help doctors learn more about treating patients with the same condition in the future. Your study doctor will tell you about new information or changes.

**WHAT OTHER OPTIONS ARE THERE?**

Your participation in this study is voluntary. Do not sign this form unless you have had the chance to ask questions and have received satisfactory answers. You may also wish to discuss this matter with a relative, a friend, or another doctor.

If you decide not to participate you will receive the standard thymoglobulin dose used at our transplant center which is 1.25mg/kg for 3 doses.

**WHAT ARE MY RIGHTS IF I TAKE PART IN THIS STUDY? CAN I STOP BEING IN THE STUDY?**

Taking part in this study is your choice. If you take part in this study you can stop at any time. Talk with your doctor before you decide to stop. It is important to tell your doctor if you are thinking about stopping, so any risks from your treatment can be evaluated. Your doctor will also want to discuss what kind of follow-up care and testing would be most helpful for you. Your doctor may stop your participation in the study at any time if he/she believes it is in your best interest; for example, if you do not follow the study rules; if you experience serious side effects; or if the study is stopped by the sponsor. If you and your doctor decide to end your study treatment, we will continue to follow your progress and continue to collect data about your progress and report that data to the sponsor. If you do not want information about your progress to continue to be shared with the sponsor, you must withdraw your consent to participate in writing as described in the paragraph below.

If you decide to withdraw your consent to participate in the study, no new data about you will be collected for study purposes unless the data concerns an adverse event (a bad side effect) related to the study. If such an adverse event occurs, we may need to review your entire medical record. All data that has already been collected for study purposes, and any new information about an adverse event related to the study, will be sent to the study sponsor.

Your decision not to participate or to stop participation in the study will not involve any penalty or loss of benefits to which you are entitled, and will not affect your access to health care at Lahey Hospital & Medical Center and its affiliates. If you do decide to withdraw your consent to participate in the study, we ask that you contact Dr. Hannah in writing to let her know that you are withdrawing consent to participate in the study. The mailing address is Hannah Gilligan, M.D., Lahey Hospital & Medical Center, 41Mall Road, Burlington, MA 01805.

If you are an employee of Lahey Hospital & Medical Center or any of its affiliated entities and do not wish to participate in the research study or wish to withdraw after signing this form, there will be no prejudice against you or influence on your employment status.

We will tell you about new information or changes in the study that may affect your health or willingness to continue in the study.

**WHAT ARE THE COSTS?**

The cost of the thymoglobulin will be billed to you or your insurance company.

The care you receive that is part of the regular care for your condition will be billed to you or your insurance carrier. Any care you receive only because you are participating in this research study will be paid for by the study. You will be responsible for your regular health insurance premiums, deductibles and co-payments. You will be responsible for all transportation costs you incur in obtaining medical treatment related to your participation in this study.

**WILL I BE PAID FOR PARTICIPATING IN THE STUDY?**

You will not be compensated, either directly or indirectly, for joining this study

**WHAT ABOUT INJURIES RELATED TO THIS RESEARCH?**

All forms of medical treatment whether routine or experimental involve some risk of injury. There may be risk associated with this study that we do not know about. In spite of all precautions, you might develop medical complications from being in this study. Immediate medically necessary treatment is available to you if you are injured as a result of this study.

If you sustain any injury during the course of the research or experience any side effect to a study drug or procedure, please contact Dr. Hannah Gilligan at 781-744-2500.

If such complications arise, the study doctor will assist you in obtaining appropriate medical treatment but this study does not provide financial assistance for medical or other injury-related costs.

You do not give up any rights to seek payment for personal injury by signing this form.

## HOW WILL INFORMATION ABOUT ME BE KEPT PRIVATE?

The Lahey Hospital & Medical Center, its affiliates complies with the Health Insurance Portability and Accountability Act (HIPAA) of 1996 and all other applicable laws that protect your privacy. Every reasonable effort is made to protect your information according to these laws. Most of the time, health information about you that is shared (disclosed) with a sponsor has your personal identifiers removed. All protected health information (PHI) that is shared (disclosed) with a sponsor is specifically listed in this informed consent document. Despite these protections, there is a possibility that health information about you could be shared (disclosed) in a way that it will no longer be protected. Our Notice of Privacy Practices (a separate document) provides more information on how we protect your information. A copy of the notice will be provided to you. Study records that identify you will be kept confidential as required by law. Federal Privacy Regulations provide safeguards for privacy, security and authorized access.

Your records may be reviewed in order to comply with federal or state regulations. Reviewers may include other doctors and healthcare professionals taking part in the study, the Food and Drug Administration (FDA), the Department of Health and Human Services (DHHS), government agencies that must receive reports about certain diseases and the Lahey Clinic, Inc. Institutional Review Board. If your research record is reviewed by any of these groups, they may also need to review your entire medical record.

Results of some of the tests you have during this research study that are not part of your regular care will not be included in your medical record.

You have the right to see and make copies of your medical records. However, if you decide to participate in this study, you will need to wait to see your research study records until the study is completed.

By signing this form, you authorize Lahey Hospital & Medical Center and its affiliates to share health information about you as described above. This authorization does not have an expiration date. Dr. Hannah Gilligan and her study team may need to correct or provide missing health information about you even after your study participation is over. The review of your medical records, described above can also take place after the study is over.

The results of this research may be published in scientific journals or presented at medical meetings, your identity will not be disclosed in these publications or presentations.

You have the right to take back (revoke) your authorization at any time by writing to Dr. Hannah Gilligan , 41 Mall Road, Burlington, MA 01805. If you take back your authorization to use health information about you, the study staff will not collect any new health information about you unless that information is about an adverse event (bad side effect) related to the study. If such an event happens the study staff, sponsor and regulatory agencies may need to review your entire medical record.

**WHOM DO I CALL IF I HAVE QUESTIONS OR PROBLEMS?**

If you have any questions concerning this study, the availability of medical care, or if you have experienced a research-related illness, injury, or emergency, contact Dr. Hannah Gilligan at 781-744-2500. This study has been reviewed and approved by the Lahey Clinic, Inc. Institutional Review Board. If you have any questions about your rights as a research subject, you may contact the Office of Research Administration at (781) 744-8027.
